# Zebrafish Prion Protein PrP2 Controls Collective Migration Process during Lateral Line Sensory System Development

**DOI:** 10.1371/journal.pone.0113331

**Published:** 2014-12-01

**Authors:** Sylvaine Huc-Brandt, Nelson Hieu, Thibaut Imberdis, Nicolas Cubedo, Michelle Silhol, Patricia L. A. Leighton, Thomas Domaschke, W. Ted Allison, Véronique Perrier, Mireille Rossel

**Affiliations:** 1 Univ. Montpellier 2, Montpellier, F-34095 France; 2 Inserm, U710, Montpellier, F-34095 France; 3 EPHE, Paris, F-75007 France; 4 Boston University School of Medicine, Department of Biochemistry, Boston, MA 02118, United States of America; 5 Center for Prions and Protein Folding Diseases, and Department of Biological Sciences, University of Alberta, Edmonton, Alberta, T6G 2E9, Canada; Université de Montpellier 2, France

## Abstract

Prion protein is involved in severe neurodegenerative disorders but its physiological role is still in debate due to an absence of major developmental defects in knockout mice. Previous reports in zebrafish indicate that the two prion genes, *PrP1* and *PrP2*, are both involved in several steps of embryonic development thus providing a unique route to discover prion protein function. Here we investigate the role of PrP2 during development of a mechano-sensory system, the posterior lateral line, using morpholino knockdown and PrP2 targeted inactivation. We confirm the efficiency of the translation blocking morpholino at the protein level. Development of the posterior lateral line is altered in *PrP2* morphants, including nerve axonal outgrowth and primordium migration defects. Reduced neuromast deposition was observed in *PrP2* morphants as well as in *PrP2^−/−^* mutants. Rosette formation defects were observed in *PrP2* morphants, strongly suggesting an abnormal primordium organization and reflecting loss of cell cohesion during migration of the primordium. In addition, the adherens junction proteins, E-cadherin and ß-catenin, were mis-localized after reduction of PrP2 expression and thus contribute to the primordium disorganization. Consequently, hair cell differentiation and number were affected and this resulted in reduced functional neuromasts. At later developmental stages, myelination of the posterior lateral line nerve was altered. Altogether, our study reports an essential role of PrP2 in collective migration process of the primordium and in neuromast formation, further implicating a role for prion protein in cell adhesion.

## Introduction

Prion protein, PrP^C^, is a conserved GPI-anchored protein that can undergo conformational changes to a ß-sheet enriched form called PrP^Sc^, which is involved in the etiology of transmissible spongiform encephalopathy (TSE). The misfolded form PrP^Sc^ is well known for its ability to recruit and template the misfolding of normal cellular PrP^C^, initiating the pathological development of the disease and TSE [1]–[3]. Furthermore, increasing data demonstrate the involvement of PrP^C^ in mediating Aß oligomer toxicity in Alzheimer's disease models [4], [5]. Aß oligomers affect the localization of PrP^C^ at the cell surface through a high affinity interaction. In addition, lack of PrP^C^ rescues memory impairment and loss of synaptic markers [1], [5], [6], [7]. Moreover prion and amyloid precursor protein have a conserved interaction, demonstrated functionally in zebrafish and at the biochemical level in humans [8].

PrP^C^ is involved in many cellular processes such as neuritic outgrowth [9], adhesion and neuronal activity [1]. PrP^C^ disruption leads to an increased sensitivity to toxins or hypoxia that results in neuronal death, reflecting a neuroprotective role for PrP^C^ [10], [11]. Mouse knockout models for the prion gene *Prnp* display normal development, metabolism and lifespan, and present with a resistance to PrP^Sc^ infection [12], [13].

In the zebrafish model, the *Prnp* gene is duplicated and the expression of the two paralagous genes *PrP1* and *PrP2* are dissociated both spatially and temporally: (i) PrP1 is expressed during early embryonic stages in the whole embryo and is down regulated before the pharyngula stage [14]; and (ii) PrP2 expression coincides with the onset of somitogenesis and is expressed in the central nervous system and cranial ganglia. In comparison with the mammalian *Prnp* gene, *PrP2* represents the closest ortholog [15].

While mouse *Prnp* gene knockout does not affect any major developmental or physiological process, *PrP1* inactivation in zebrafish results in a dramatic phenotype with cellular movement defects and early embryonic lethality [8], [14], [16]. Such severe phenotypes have been linked to the loss of blastomere cell adhesion and in particular to the decreased stability of adherens junctions. *PrP2* inactivation leads to nervous system malformations that affect the anterior part of the neural tube, essentially the telencephalic, midbrain and hindbrain regions [14], [17]. However, discrepancies have been observed following *PrP2* gene inactivation using gene targeting, as mutant embryos or larvae show no developmental abnormalities but impaired NMDA receptor regulation [18]. Whether the *PrP2* gene is required in nervous system development is still in debate and morpholino-mediated inactivation has to be carefully evaluated.

To clarify the role of PrP2, we took advantage of the well-characterized mechano-sensory system, the zebrafish posterior lateral line (PLL). The PLL ganglion displays a strong expression of PrP2 as early as 30 hours post-fertilization (hpf) and at later developmental stages *PrP2* mRNA is observed in the differentiated sensory organs, including in the neuromasts and in its differentiated hair cells [14], [19].

The PLL provides a powerful *in vivo* model to study multiple cellular processes such as cell migration, axonal outgrowth and differentiation process. PLL development relies on the migration of the primordium, a cohesive group of cells that is organized and polarized throughout the migration process. Sensory organs called neuromasts are organized in a stereotype pattern along the PLL at the body surface. Hair cells positioned at the center of each neuromast register and measure water movements and are homologs of mammalian inner ear hair cells [20].

In the present study, using morpholino knockdown, we performed partial gene inactivation and demonstrated that PrP2 is required for the development of the PLL. Transient inactivation of *PrP2* gene induced abnormal neuromast deposition, as well as defects in the PLL nerve trajectories. Comparison with loss of function *PrP2* mutant confirmed abnormal neuromast positioning and partial disorganization of the primordium. Furthermore, *in vivo* analysis of the primordium development revealed an abnormal migration with premature arrest. Our results suggest that PrP2 is necessary for the formation and/or stabilization of the presumptive neuromast, a pseudo-epithelial rosette, during the migration process.

## Results

### Reduced axonal outgrowth and neuromast number in PrP2 morphants

The *PrP2* gene is expressed in the central nervous system and the cranial ganglia starting during somitogenesis and peaks at 30 hpf [14]. As prion protein is involved in neuritic outgrowth [21], we examined the development of PLL nerve and neuromasts after morpholino inactivation of *PrP2*.

**Figure 1:**
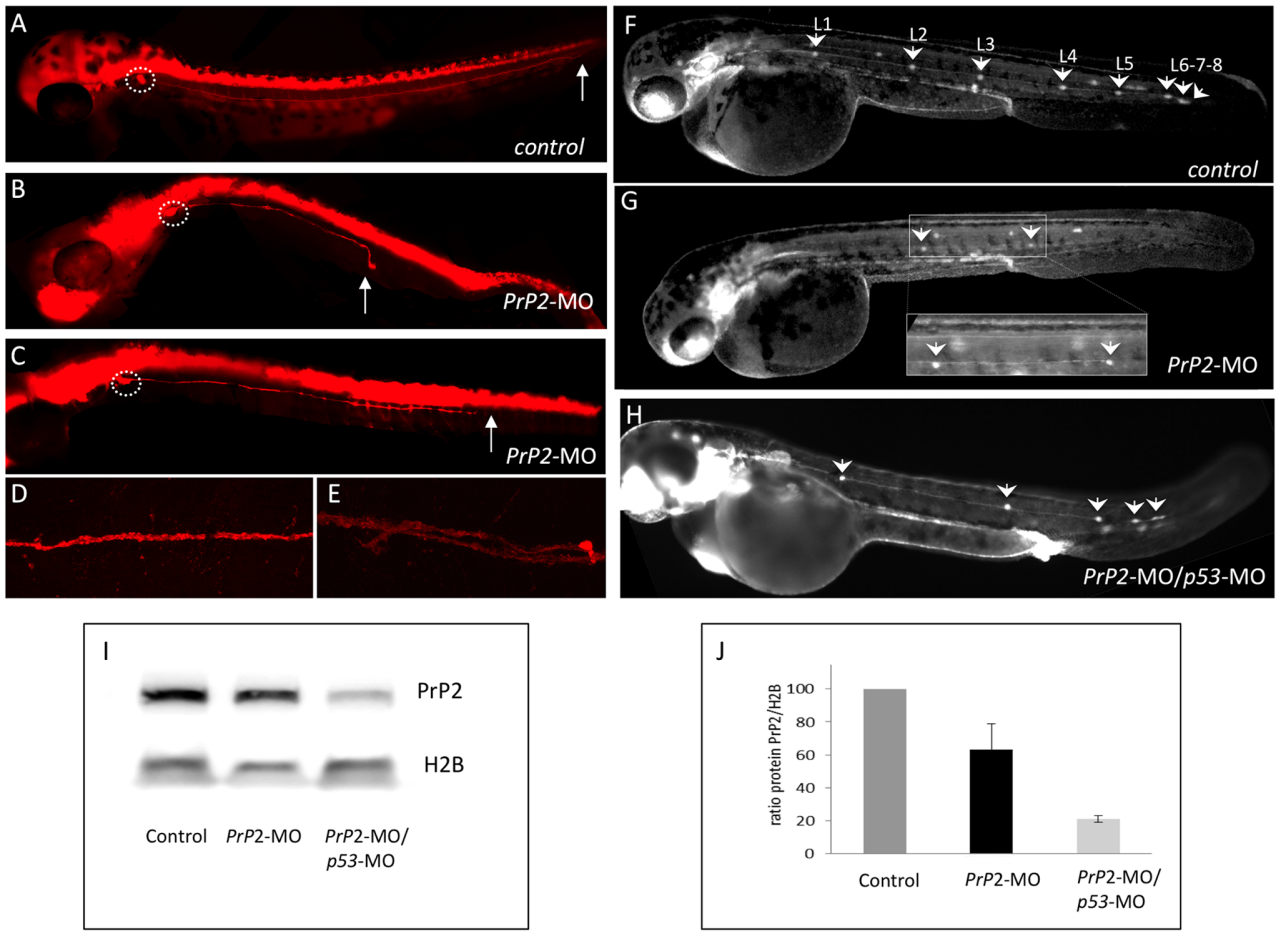
PrP2 decrease expression results in abnormal PLL development. **A**. In control embryos at 48 hours post fertilization (hpf) from *nbt-dsred* line that labels neurons and axons, the PLL nerve develops from the PLL ganglia until the tip of the tail (arrow). **B–C**. In *PrP2*-MO embryos, a range of defects for the PLL nerve is observed with premature arrest (B, C). **D**. Control PLL nerve fibers are tightly while *PrP2*-MO fibers exhibit abnormal branches were observed in severe cases (**E**). **F**. In control *claudinB-GFP* embryos at 48 hpf all derivatives issuing from the primordium express GFP and appear normal. Five regularly spaced neuromasts are present, with 3 terminal neuromasts at the tip of the tail. Due to embryo transparency, the neuromasts on the other side of the embryo are also visible. In *PrP2*-MO (**G**) and *PrP2*-MO/*p53*-MO (**H**) injected embryos (in order to avoid off-target defects), neuromast numbers are reduced, and often irregularly spaced. **I–J**. Western blot analysis of PrP2 expression using SAF84 antibody shows a decrease of the PrP2 protein after morpholino injections (n = 3 independent experiments).

Previous studies have shown major cell death within the head, especially in the nervous system, following morpholino-mediated *PrP2* inactivation [14], [17]. We also observed such defects with high morpholino concentrations (data not shown). To avoid the typical non-specific effects of morpholinos as previously described [22], we used a range of concentrations that did not induce major cell death and allowed normal embryo development (Figure 1). Two novel morpholinos were tested that target the initiation codon (MO1) or the 5′UTR sequence (MO2) and the same phenotypes were observed for each. Therefore *PrP2*-MOs were injected at a low concentration i.e. 0.3 mM (10 ng) allowing normal, or nearly normal, body development and observation of lateral line phenotypes (if any).

During normal development, tightly fasciculated axons form the posterior lateral line nerve and innervate the developing neuromasts. We first examined PLL nerve development at 48 hpf using the *nbt-dsred* line that labels neurons and axons. Axons from the PLL ganglion neurons reach the tail extremity at 48 hpf in control embryos (Figure 1A). In *PrP2* morphants (*PrP2*-MO), the nerve was always shorter (n = 150, Figure 1B, C) and in some cases presented with defasciculated axons (Figure 1D, E). The ganglion size and neuron number were similar to control embryos, indicating that the ganglion forms normally (Figure 1A–C and data not shown). The PLL nerve develops in close contact with the primordium that migrates and deposits pro-neuromasts along the midline body; thus the shortening of the nerve and abnormal pathway suggested a defect in primordium migration.

Therefore, we studied the organization of the neuromasts in *PrP2* morphants. As the PLL primordium migrates, it normally deposits six to eight proneuromasts (L1 to L8) along the horizontal myoseptum and spaced at regular intervals [23], . By 48 hpf, the primordium has reached the tip of the tail where it stops migrating and gives rise to three terminal neuromasts (L6, L7 and L8) [25], [26] (Figure 1F). To examine the positions and numbers of neuromasts at 48 hpf, we used the *claudinB-GFP* transgenic line, which labels all PLL cells: from the primordium to neuromasts and interneuromastic cells [27]. Compared to control embryos (mean ±sem: 8±0.7, n = 150) (Figure 1F), the neuromast number was decreased in *PrP2*-MO (mean ±sem: 5.3±0.3, n = 150, p<0.001) (Figure 1G) and in addition to this reduction, their organization was severely altered. The efficiency of the morpholinos was assessed with Western blot analysis, revealing a decrease of 30% relative to controls (Figure 1I, J). Moreover, *p53*-morpholino was injected in combination with *PrP2* morpholinos at two concentrations (0.3 and 0.7 mM) to restore any unspecific defects linked to *p53* overexpression [17], [18]. We found a reduced PLL phenotype with the ratio of 0.3/0.7 *PrP2*-MO/*p53-*MO (Figure 1H). We used this latter condition to compare the total number of neuromast and their positions relative to the somites.

**Figure 2:**
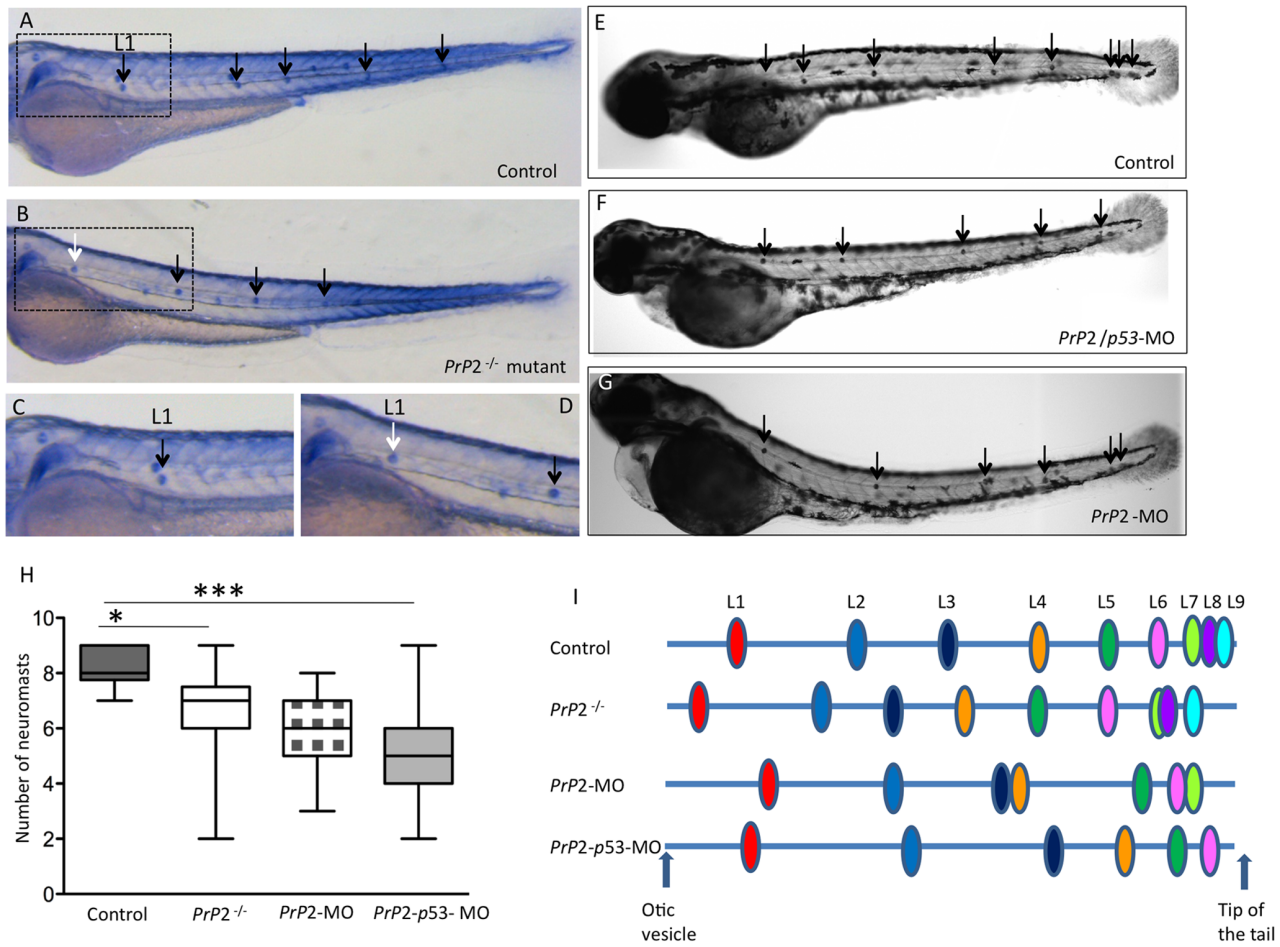
PrP2 is involved in PLL development. **A, C.** Alkaline phosphatase staining of the trunk neuromasts (arrows) in wild type embryos at 72 hpf. **C**. High magnification of boxed area in A. **B, D.** In *Prp2^−/−^* mutant, less neuromasts are visible with the first neuromast displaced anteriorly (**B**, white arrow) **D**. High magnification of boxed area in B. **E–G.** In Control **(E)**, *PrP2*-MO/*p53*-MO (**F**) and *PrP2*-MO (**G**), the total neuromast number is decreased in morphants. **H.** Quantification of total neuromast number shows a significant decrease in morphants and mutants compared to control. **I.** Neuromast position along the somite axis, scheme adapted from [28]. In *Prp2^−/−^* mutant, L1 is retrieved anteriorly to the normal L1 position while in morphants, the present neuromasts are displaced posteriorly. *: p<0.05, ***: p<0.001.

The positions of the neuromasts were also analyzed in older embryos, at 72 hpf, to address any possible delay in development (Figure 2). After alkaline phosphatase staining, differentiated neuromasts were assessed in *PrP2* ^−/−^, *PrP2*-MO and *PrP2*-MO/*p53-*MO (Figure 2A–G). In *PrP2* ^−/−^ fish significantly fewer neuromasts were observed compared to control wild type embryos (Figure 2A–D, H, mean ±sem: 6.6±0.2, n = 45, compare to 8.1±0.2, n = 40, p<0.05). Quantification in morphants revealed comparable decreases and significantly fewer neuromasts compared to control embryos (Figure 2F, G, mean ±sem: 5.7±0.1, n = 59, p<0.001 and 5.4±0.3, n = 45, p<0.001, respectively for *PrP2*-MO and *PrP2*-MO/*p53-*MO).

Interestingly, instead of the regularly spaced neuromasts in the trunk region of control embryos (as represented in Figure 2I [28]), *PrP2* ^−/−^ mutant embryos deposited the first neuromast prematurely (L1, localized close to the ear) and the following neuromast was typically found at anterior positions, whereas the last three neuromasts were often absent (Figure 2A–D, I). In addition, in *PrP2*-MO and *PrP2*-MO/*p53-*MO embryos, the neuromasts were positioned in an erratic pattern (Figure 2I): in most cases, the L2 and further positions were displaced posteriorly with the last three neuromasts often missing (Figure 2F, G, I).

To assess the phenotype specificity, we made several attempts to rescue the PrP2 defects. We used mRNA injection from either zebrafish *PrP2* or mammalian *Prnp* cDNAs (mouse or human). As previously reported [8], [14], we confirmed the absence of rescue of the PLL phenotype (data not shown). As such results are not necessarily informative, because mRNA delivery in morphants can also fail to rescue phenotypes for technical reasons (e.g. mRNA stability), we instead focused on assessing reagent specificity by comparing the results of three independent PrP2 loss-of-function methods.

In conclusion, we have studied the effect of *PrP2* inactivation by different means and we observe similar defects (i) with two different morpholinos, or (ii) with stable targeted mutation. Our results strongly suggest that *PrP2* inactivation leads to PLL abnormal development with a reduction of total neuromast number and perturbed distribution along the body axis.

### PrP2 expression in the primordium and neuromasts

To further investigate the role of PrP2 in PLL development, it was important to analyze PrP2 expression within the primordium, since its expression was already demonstrated in the neuromasts [19].

**Figure 3:**
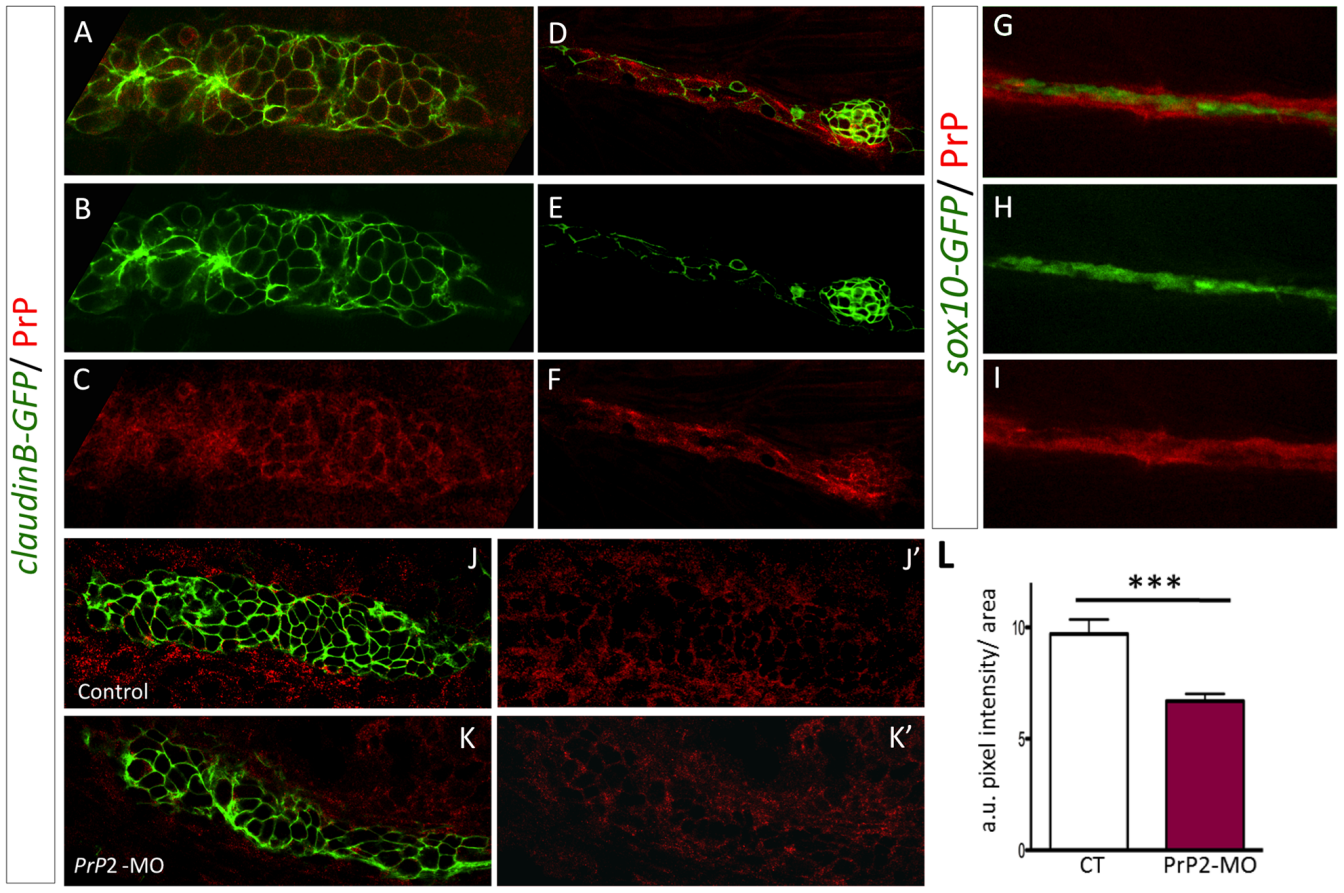
PrP2 is expressed in the primordium. **A–C.** Immunofluorescence on 30 hpf *claudinB-GFP* embryos using anti-PrP^C^ antibodies, revealed PrP expression at the membrane of primordium cells during the migration process. **D–F**. Neuromast cells as well as interneuromastic cells are positive for PrP2. What is represented by the arrows here? **G–I**. PrP immunofluorescence on 48 hpf sox10-GFP embryos, showed that Schwann cells are included in PrP expression area. **J, J**′. Expression level of PrP2 within the primordium in control embryos. **K, K′**. Expression level of PrP2 within the primordium in *Prp2*-MO. **L**. Quantification analysis using pixel intensity measurement with ImageJ software, within the primordium areas, reveals a significant decrease of PrP2 expression level in *Prp2*-MO. a.u.: arbitrary unit of pixel intensity, area: primordium anlage (*** p<0.001, Mann-Whitney test, n = 10).

For this purpose, we selected several monoclonal antibodies directed against Scrapie Associated Fibrils (SAF) purified from prion infected hamster brain and known to recognize the human proteins PrP^C^ and PrP^Sc^. Sequence alignments for the zebrafish and mammalian proteins revealed a high conserved homology for one region (126–160 aa), thus we tested 6 different monoclonal antibodies targeting this region. Only one clone gave a specific signal, the clone SAF84, whose targeted epitope overlaps with the 126–164 human aa sequence (Figure S1) [29]. Monoclonal antibodies directed against the 142–160 aa PrP^C^ sequence did not give any encouraging results. Specificity of the SAF84 antibody was supported by Western blot analysis showing a decrease in the SAF84 labeling when PrP2 levels were reduced (Figure 1I, J). Further, we determined the effectiveness of *PrP2*-MO on protein levels using immunofluorescence. At 30 and 48 hpf, we observed a reduction of PrP2 labeling in embryonic tissues (Figure 3K, K′) compared to control (Figure 3J, J′). In morphants, quantification of PrP2 expression in the primordium revealed a significant decrease of 32% consistent with the decrease observed with Western blot (Figure 3L, control n = 5, morphants n = 10). Our results indicate that a mild decrease in PrP2 protein lead to an impairment of PLL development.

We performed immunolabeling using SAF84 antibodies at 30 hpf, when the primordium is migrating, and at 48 hpf after neuromast deposition. PrP2 expression within the primordium (Figure 3A–C) in *claudinB-GFP* embryos demonstrated its distribution in membranes and coincided with *claudinB-GFP* signal. PrP2 expression was also present in interneuromastic cells that are deposited along the migratory pathway. In neuromasts, we observed PrP2 expression in all cell types, namely hair cells, support cells and mantle cells (Figure 3D–F). In addition, the PrP2 expression area included glial cells which was confirmed by the presence of *sox10-GFP* cells within the PrP2 positive area (Figure 3G–I).

In conclusion, we describe the presence of PrP2 protein within the precursors cells (primordium) as well as in the sensory organs of the PLL. This expression pattern is consistent with our observations following inactivation of *PrP2*: abnormal migration of the primordium associated with defects in PLL nerve and neuromast deposition (Figure 1 and Figure 2).

### Premature arrest of primordium migration and cell cohesion deficiency

During normal development, the migrating PLL primordium shows a segmental pre-pattern along the axis of migration, with cells in the trailing region organized into distinct rosettes of cells, each corresponding to a proneuromast that is later deposited, and the leading part (approximately one third of the primordium) remaining un-patterned [30]. Cells behind the leading edge become columnar epithelia and undergo apico-basal polarization before the constriction of actin-rich membranes and the enrichment of apical junction proteins into focal points to form rosettes.

**Figure 4:**
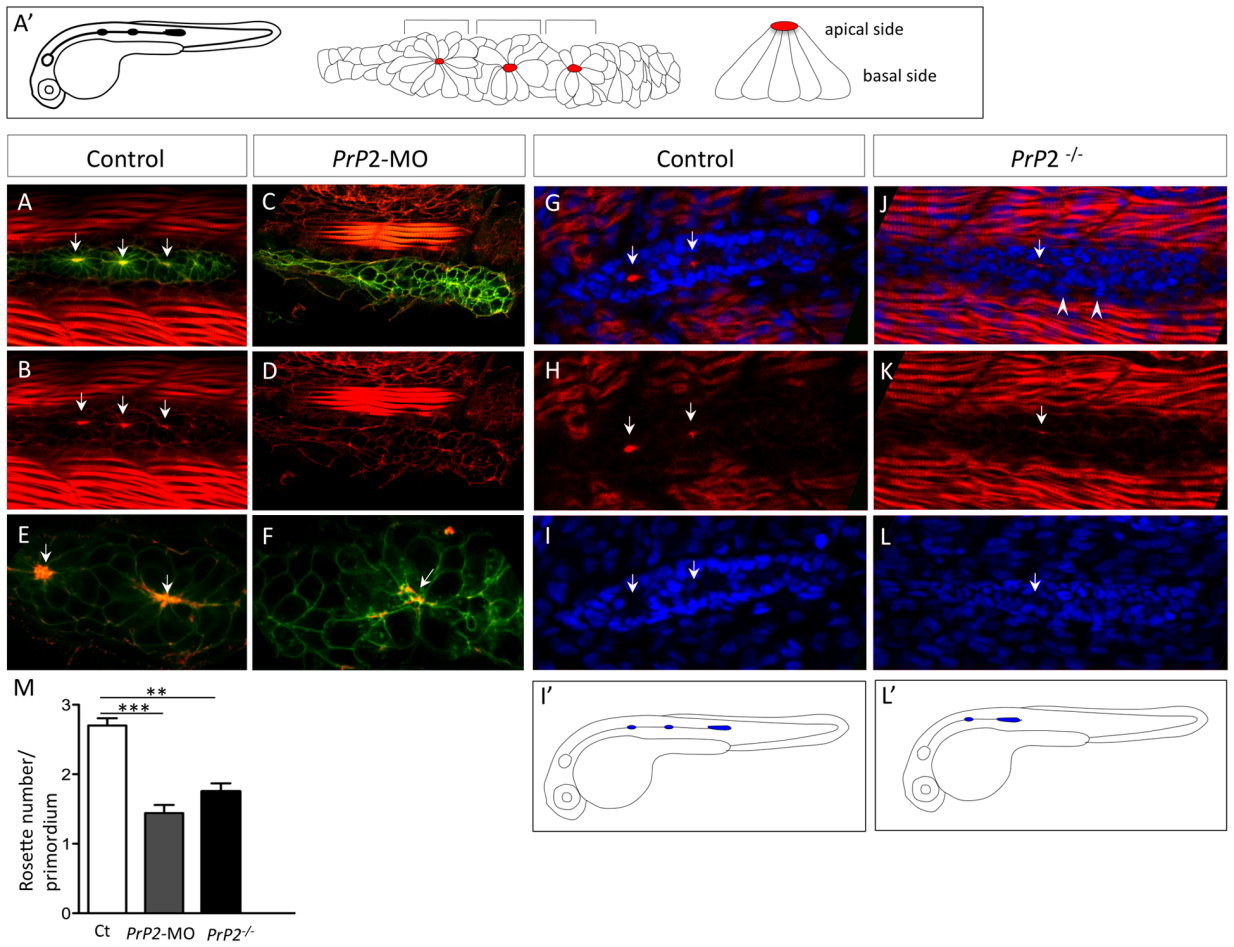
Primodium disorganisation and absence of rosette formation. **A′.** Schematic representation of a normal *claudinB-GFP* embryo at 30 hpf and detailed organization of the primordium with rosette structure. Red spot indicates a normal concentration point of actin. **A, B.** Phalloidin staining (Phalloidin-TRITC) in control embryo *claudinB-GFP*, at 30 hpf, is observed in muscle cells and within the primodium at the center of the rosette (arrows), on the apical side. **C, D.** Phalloidin-TRITC staining in *PrP2*-MO, no rosette structure is observed and no actin concentration is found. **E, F.** Higher magnification shows the co-localization of central actin concentration with *claudinB-GFP* at the rosette center in control. In morphants, cell disorganization is observed and no actin concentration is observed associated with the absence of a rosette. **G–I.** Phalloidin staining and DAPI nuclei labeling highlight the primordium and rosette center (arrows) in control embryos. **J–L.** In *PrP2^−/−^* mutants, actin apical localization in rosette was severely reduced or barely detectable (arrow) and primordium organization at the periphery was impaired: loose cells were visible on the border (arrowheads). **I′, L′.** In *PrP2^−/−^* mutant, the primordium position was often delayed and the first neuromast deposited close to the ear. **M**. Quantification of rosette number was established in control (n = 20), *PrP2*-MO (n = 84) and *PrP2^−/−^* mutant (n = 28) using actin staining at the center, **: p<0.01, ***: p<0.001, Student t test. See also associated Movies S1–S5.

As previous studies demonstrated the importance of the rosette formation for the primordium cohesion and migration [30], [31], we therefore assessed whether the presence of cellular rosettes within the PLL primordium was affected in embryos with decreased PrP2 expression (Figure 4). Quantifications of cellular rosettes were performed based on actin focal points within the PLL primordium [32]. Two to three rosette-like proneuromast can be morphologically identified within wild-type primordia (Figure 4A, B, E), but no such organization was visible in 60% of *PrP2*-MO embryos aged between 28 and 36 hpf (n = 61/102) (Figure 4C, D, F, M). The absence of actin concentration at the rosette center might reflect a loss of apical junction that promotes the apical constriction of the pro-neuromast cells in *PrP2*-MO (Figure 4F, M, number of rosette: 1.4±0.1, n = 84). At 30–31 hpf, in *PrP2* ^−/−^ mutant and wild type embryos, we performed a similar analysis using DAPI nuclei and phalloidin staining. In controls, the primordium edges are clearly visible with DAPI staining and the actin focal points are well defined (Fig. 4G–I, Movies S1, S2). In absence of PrP2, the primordium organization was not as well delineated as in the control, with loose cells at the periphery of the primordium (Figure 4J, arrowheads). Moreover, the apical center of the rosette was not prominent and actin labeling was clearly reduced (compare Figure 4G, H and 4J, K, Movies S2, S3). Quantifications indicate a significant decrease of rosette number in *PrP2* ^−/−^ mutants compared to control embryos (Figure 4M, number of rosette: 1.7±0.1 versus 2.7±0.1 for controls, p<0.01, n = 20). Although *PrP2* ^−/−^ and control embryos were at the same developmental stage, the primordium progression was always delayed (Figure 4I′, L′), and its shape often elongated (Figure 4J, Movies S4, S5).

**Figure 5:**
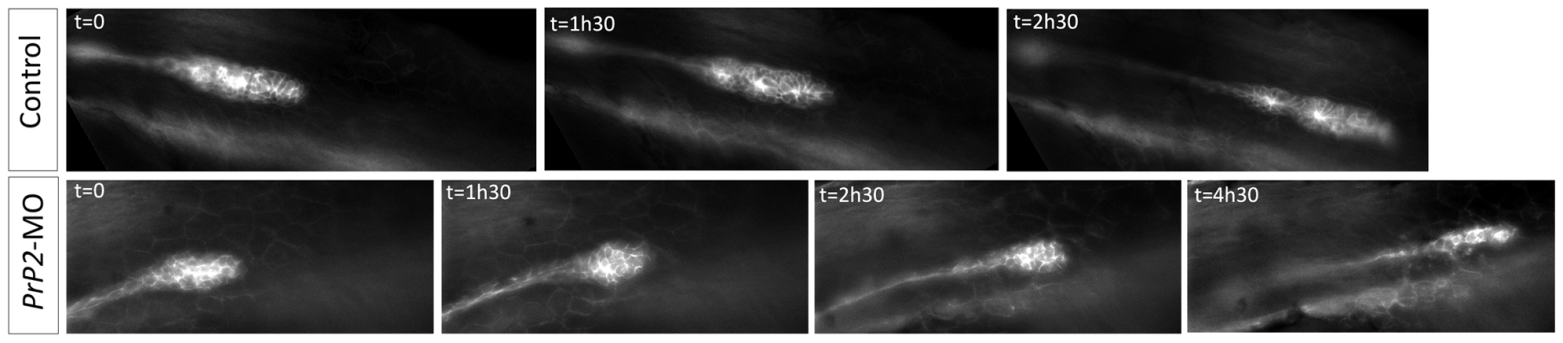
Loss of cell-cell contact and disorganization of collective migration of the primordium. Select time points from time-lapse recording of primordium migration in *claudinB-GFP* embryos. In control embryos, primordium migration is continuous, with a neuromast deposition (time point 2h30) and out of view at the time point 4h30 (not shown). In contrast in one *PrP2* morphant example, representative of the observed phenotypes, primordium migration shows a progressive rounded shape and arrest. See also Movies S6 and S7.

To understand the role of PrP2 during PLL development, we followed the primordium migration at earlier stages using time-lapse analysis on *claudinB-GFP* embryos. In control embryos, the well-organized collective migration of the primordium corresponds to a synchronous and smooth movement (Figure 5, Movie S6). In *PrP2*-MO, the primordium migration was irregular and rapidly became rounded and eventually stopped (Figure 5, Movie S7). Because PrP^C^ and PrP1 have been shown to be involved in cell-cell contact and cell adhesion during gastrulation [8], [14], we next examined the cohesive properties of primordium cells.

### Adherens junction disorganization in primordium of PrP2 morphant

The adherens junction component E-cadherin is necessary for the proper cohesion and apical polarity of the rosette-like structure [28], [33]. For example, loss of E-cadherin expression is observed when PLL is disrupted including when notch signaling fails and *atoh1a* expression expands in mind bomb mutants (*mib1*). Disruption of E-cadherin is associated with fragmentation of the primordium and proneuromasts, suggesting a role in primordium cohesion [28]. As PrP1 protein can mediate E-cadherin membrane location [14], [16], we examined the expression and membrane location of E-cadherin and the associated protein beta-catenin.

**Figure 6:**
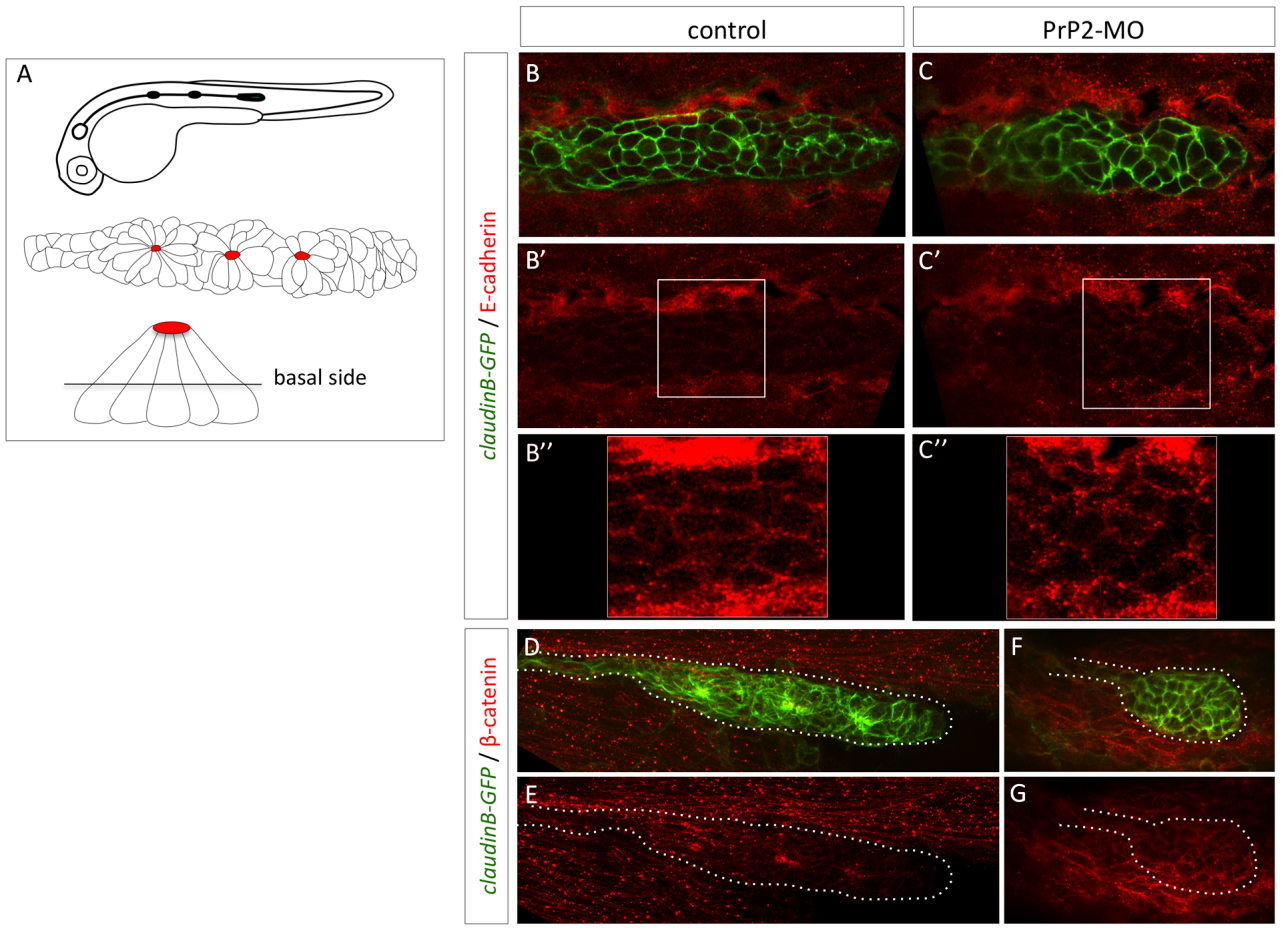
Delocalisation of E-cadherin and beta-catenin in primordium cells in absence of PrP2. **A**. Schematic representation of *claudinB-GFP* embryo at 30 hpf, primordium structure, and transversal view of a rosette showing the level of confocal focus plan. **B–B″**. In control embryos at 30 hpf, E-cadherin is expressed at the membrane level at the basal side of the primordium. **C–C″.** In morphant embryos, E-cadherin is observed in the cytoplasm and cellular membrane at basal and apical levels. The staining pattern is more punctuated in morphant compared to control. **D, E.** In control embryos, beta catenin expression is observed at the membrane level and at the center of rosette structures. **F, G**. In morphant, small rounded primordium shows a membrane homogeneous pattern with no rosette.

In control primordium, E-cadherin expression was localized uniformly throughout the entire PLL primordium at all intercellular membranes and was enriched at the periphery [28], [34] (Figure 6A, B′, B″). This contrasted with the primordium cells in *PrP2*-MO embryos, where a delocalization of the membrane E-cadherin to a cytoplasmic localization was apparent (Figure 6C, C′, C″), the staining pattern is abnormally punctuated. Knockdown of *PrP2* also resulted in the alteration of the adherens junction organization throughout baso-apical axis of the primordium. Beta-catenin was also analyzed as another component of the adherens junctions. In control embryos, beta-catenin was concentrated at the apical focal point at the rosette center (Figure 6D, E) [32] while in *PrP2*-MO, beta-catenin was localized at the membrane of the rounded primordium, devoid of rosette structure (Figure 6F,G).

Altogether, the alterations of F-actin, E-cadherin and beta-catenin localization strongly suggest that PrP2 is involved in the maintenance of adherens junctions that contribute to the dynamic organization of the primordium. Consequently, failures in rosette formation apparently produce defects in neuromast deposition and maturation.

### PrP2 role in hair cell development

During neuromast development, rosette formation is tightly controlled through lateral inhibition mechanism at the rosette center that determine hair cell fate [25], [28], [30], [31], [33], [34]. Next, we investigated if the rosette disorganization we observed might also influence the number of hair cells within a neuromast.

**Figure 7:**
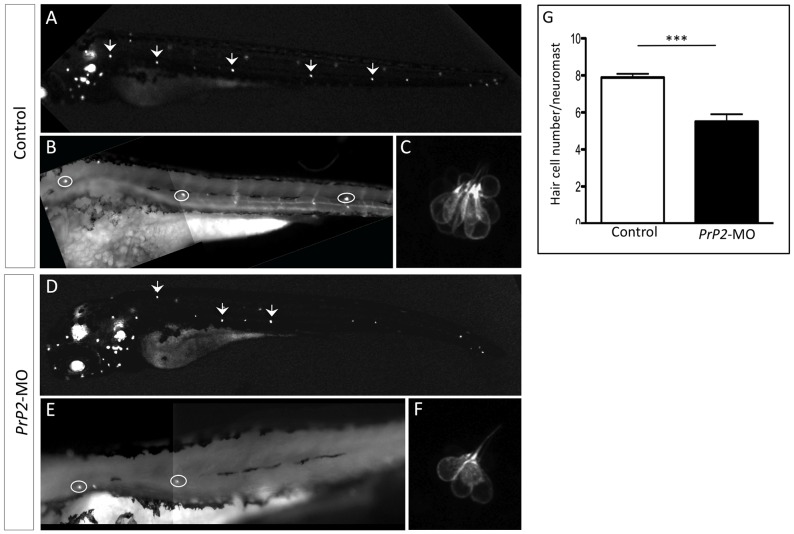
Decreased hair cell number of PLL neuromast in *PrP2*-MO. **A–C.** In control embryos at 48 hpf, *Brn3-GFP* fluorescence labels hair cells of neuromasts and ear. High magnification of the first 3 neuromasts and zoom of the first neuromast show 8 hair cells. **D–F**. Morphants displays reduced number of neuromasts. High magnification shows smaller hair cells number. **G**. Quantification indicates significant reduction of hair cells/neuromast, independently of the neuromast position (mean hair cell number: 5.5±0.4, n = 36, compare to control 7.9±0.2, n = 20, p<0.001).

Using the *Brn3::GFP* line that labels hair cells, an average of eight hair cells per neuromast was observed in control embryos at 3 dpf. A decrease in the number of hair cells was found in *PrP2*-MO, and this was independent of the neuromast organization: quantitative analysis showed that most of the neuromasts display 4 to 8 hair cells (Figure 7, mean hair cell number: 5.5±0.4, n = 36, compare to control, 7.9±0.2, n = 20, p<0.001). We conclude that PrP2 is important for hair cell fate through the epithelial organization of rosette structure.

### PrP2 role in Schwann cell maturation

**Figure 8:**
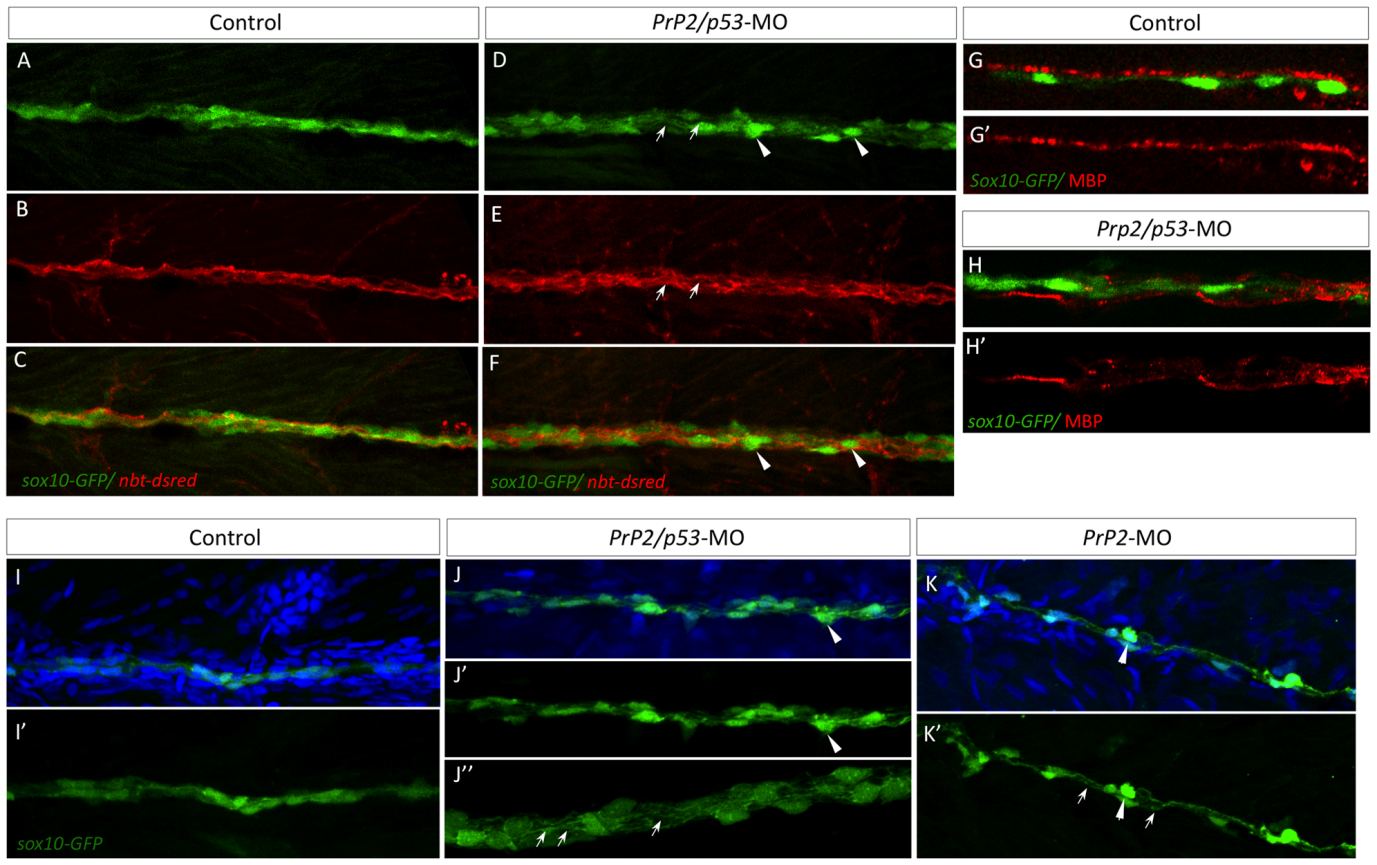
Loss of PLL nerve fasciculation and associated myelination in absence of PrP2. **A–C**. In control larvae at 5 dpf, *sox10-GFP* cells are tightly organized along the PLL nerve (*nbt-dsred*). **D–F**. Double morphant *PrP2/p53* displayed an enlarged PLL nerve (with defasciculated axons, arrows) and rounded Schwann cells (arrowheads). **G–G**′. In 5 dpf larvae MBP labeling is observed in close apposition to *sox10-GFP* cells and formed a homogeneous line. **H–H′**. MBP labeling is altered and partially missing while Schwann cells are disorganized. **I–I′**. At 7 dpf, control larvae display tightly and regularly organized *sox10-GFP* positive cells. **J–K′**. In *PrP2/p53* morphants (J–J″), loosened Schwann cell processes are observed (arrows) as well as rounded cells (arrowheads) in *PrP2* morphants (K–K′). Results obtained from five independent experiments (n = 160 embryos).

We next asked if PrP2 is necessary for Schwann cell development and proper myelination of the posterior lateral line nerve. It has been shown previously in *Prnp* mouse models that the crosstalk between PrP^C^ expression in axons and Schwann cells was required for myelin maintenance [35]. Using a *sox10-GFP* line, we followed Schwann cell development and organization in *PrP2*-MO and *PrP2/p53*-MO embryos and larvae up to 7 dpf. During embryogenesis, Schwann cells migrate along the PLL nerve during PLL development and then proliferate [36], [37]. Up to 3 dpf, control and *PrP2* morphant embryos were similar with *sox10-GFP* positive cells following and covering the PLL nerve (data not shown). At 5 (and 6) dpf, we observed a disorganization of Schwann cells in *PrP2*-MO and *PrP2/p53*-MO (Figure 8). The tight pattern of Schwann cells and their overlapping extension processes (Figure 8, A–C) were lost in morphants: abnormal rounded Schwann cells were observed along the axonal bundles labeled with neural beta tubulin (*nbt-dsred* line) (Figure 8, D–F) Moreover, axons frequently showed defasciculation that was also associated with Schwann cell loosened processes still associated with axons (Figure 8 D–F, arrows). Myelination is part of the differentiation process of Schwann cells and mbp is a marker of myelinating glia. MBP expression was localized in close apposition with *sox10-GFP* labeling in control 5dpf embryos (Figure 8 G,G′) while in double morphants, MBP labeling was discontinuous and partially dissociated from *sox10-GFP* positive cells (Figure 8 H,H′).

Similar defects were observed with both *PrP2*-MO and *PrP2/p53*-MO at 7dpf (Figure 8, D–F 5dpf compared to J–K′, 7dpf, arrows J′). PrP2 is required for normal axonal fasciculation of PLL nerve as well as glial cell close association with axons.

## Discussion

Recent studies highlight the developmental role of *PrP* genes during zebrafish development. Two genes have been characterized, *PrP1* and *PrP2*. *PrP1* is essential during early embryonic development and regulates cell adhesion during gastrulation [14], [19]. *PrP2* is expressed in the nervous system and its inactivation results in a wide range of phenotypes: from severe head and nervous system malformations to normal embryos [10], [12], [13]. Recent data, with targeted Zinc-Finger Nuclease-mediated inactivation, results in a stable mutant line (*PrP2^ua5001/ua5001^)*. This mutation gives rise to a short PrP2 truncated protein. Homozygous mutant fish have no major nervous system malformations but defects in neuron excitability [18].

The role of PrP2 has been described by these three studies that have in common the absence of rescue using either zebrafish *PrP2*, human or mouse *Prnp* mRNA injections [14], [17], [18]. mRNA delivery in morphants can also fail to rescue phenotypes for technical reasons depending on the mRNA stability. Such observation raises possible specificity problems of the morpholino inactivation. Using low but effective morpholino concentrations and *p53*-MO double inactivation in order to reduce off-target defects, we show that PrP2 is involved in PLL development. Although we failed to rescue the phenotype, we address the protein expression level in embryos and show that in morphants, PrP2 protein is reduced as measured by Western blot and immunofluorescence. In addition, the comparable phenotypes obtained with the two morpholinos and the loss of function *PrP2* mutant support a specific effect of the morpholino knockdown.

### PrP2 and cell adhesion/cohesion in PLL primordium

In the present study we show for the first time the expression and function of PrP2 protein during PLL development. *PrP2* expression at the mRNA level is observed in the central nervous system at the pharyngula stages and in two cranial ganglia: the trigeminal and the posterior lateral line ganglia [14], [18]. *PrP2* mRNA localization in the PLL system has been reported in neuromasts at 3 dpf and later [19]. The protein expression is present as early as 24 hpf, after the primordium has started its migration. The interneuromastic cells as well as Schwann cells are also positive for PrP2 protein expression, which is consistent with the expression of PrP^C^ in the peripheral nervous system. The pattern of expression corresponds to the membrane labeling of the primordium and neuromasts cells, in line with the GPI-linked structure of the protein.

During migration, the primordium maintains a remarkable level of cohesion, and dynamic regulation of cell-cell adhesion between primordium cells is crucial for lateral line morphogenesis. Differential adhesion during lateral line morphogenesis is a largely unexplored area of research. Here we provide evidence for the involvement of PrP2 in the cohesive migration by mediated adherens junction formation at the cell-cell contact. E-cadherin delocalization from the cell membrane is observed after PrP2 decrease. A role of PrP2 in regulation of cell junction is consistent (i) with the role of PrP1 in E-cadherin localization regulation, and (ii) with recent data on the ability of *PrP2* mRNA to rescue *PrP1*-MO phenotypes [14]. However, analysis of functional domains revealed clustering of PrP1 at cell-cell contact while PrP2 was continuously expressed at the cell membrane, as was mouse PrP^C^ [16].

Our findings are consistent with recent data on human A431 epidermoid carcinoma cells where down regulation of PrP^C^ results in less well-defined adherens junctions, and reduced basal to apical cell movement [16]. Interestingly, loss of primordium cell cohesion similar to what we report here has been reported in mind bomb 1 (mib1) mutant zebrafish: *mib1* mutants have a severe loss of Notch signaling and a loss of cohesion within PLL cell primordium that results in premature arrest of migration and disorganization of neuromast deposition, comparable to *PrP2*-MO phenotype [33]. A down regulation of expression of the E-cadherin was observed in mib1^m178^ mutants and linked to an expanded atoh1a expression in both the primordium and deposited neuromasts. In addition, the recovery of E-cadherin expression was associated with improved cell cohesion, suggesting that the reduction in E-cadherin contributes to the fragmentation of the primordium [28]. Altogether, the present study provides evidence for the role of PrP2 in the cohesiveness of the primordium in interaction with E-cadherin, supporting an ancient role for prion protein in cell adhesion that is retained by mammalian as well as both paralogs of the zebrafish gene products.

### PrP2 and hair cell differentiation

We observed a significant decrease in hair cell number in the PLL neuromasts of *PrP2*-MO. In control embryos, hair cell number increases overtime: neuromasts with 8 hair cells at 72 hpf will have 12 hair cells few days later. In *PrP2*-MO, neuromasts has a fixed hair cell number that did not change at later developmental stages. The effect of PrP2 knockdown on hair cell development is probably a secondary consequence linked to abnormal organization of the rosette and primordium.

Prion protein is known to bind copper cation through a specific motif [38]. In cell culture models, PrP^C^ transcript and protein levels were consistently elevated upon copper deficiency and the up-regulated PrP^C^ has been demonstrated to enhance the cell copper uptake ability. In zebrafish, the corresponding copper binding domain within the octa-repeat region lacks the histidine residues necessary for copper interaction. Neuromast hair cells are highly sensitive to copper toxicity and able to regenerate, upon retrieval of copper solution, the missing hair cells up to the same number. PrP2 decrease induces a primary failure in neuromast formation that leads to a decrease in support cells and hair cell precursors but does not impair hair cell regeneration capability (data not shown).

### PLL as a model to study prion and APP interaction

Here we show that the physiological function of PrP2 includes a role in the development of a mechano-sensory system, the posterior lateral line. In mammals, *Prnp* inactivation led to chronic demyelinating polyneuropathy and was due to the absence of PrP^C^ in neurons of peripheral nerves [35]. Here we show that PrP2 is necessary to maintain the nerve integrity through fasciculation process and myelination of individual axons. The first steps of Schwann cells development occurs normally (migration, proliferation and myelination) but at later stages, axonal bundles are abnormally loose and Schwann cells are destabilized.

Recently, zebrafish mutants for APP processing enzymes, BACE1 and BACE2 were generated [39]. BACE1 mutants also showed defects in PLL development with hypomyelination due to Schwann cell failure of myelination and consequently increased neuromast numbers [39]. Since PrP^C^ regulates BACE1 activity and APP processing, it will be interesting to test a functional link between the two proteins in the PLL system [40], [41]. In addition, PrP1 has been shown to interact with zebrafish *APP* genes in central nervous system, thus it will be important to address the question with *PrP2* and zebrafish APP genes, *appa* and *appb*, especially within the lateral line system [8].

It is now established that PrP^C^ binds Aß oligomers and participates to amyloid toxicity in Alzheimer disease. Aß oligomers regulate the membrane abundance of PrP^C^ by inhibiting PrP^C^ endocytosis and, in addition, induce its clustering at the cell surface [6]. These interactions can be studied in zebrafish since the different tools have been validated.

In conclusion, we show not only that PrP2 is necessary for PLL development but also that our zebrafish model is valuable to study the neuroprotective function of prion protein in interaction with APP protein processing.

## Material and Methods

### Ethics statement

All zebrafish husbandry and experimental procedures complied with the INSERM and Montpellier University animal welfare guidelines and register under the agreement # A34-172-37. The present study was approved by INSERM, Montpellier University and the European Convention for the Protection of Animals used for Experimental and Scientific Purposes. Following the European Directive 2010/63/EU and French Directive R-214-87 (2013), zebrafish embryos ranging from 2 to 3 days post fertilization are not considered for animal experimentation and ethics approval. Experimental procedures conducted at the University of Alberta complied with the University of Alberta Animal Care and Use Committee guidelines under the directives of the Canadian Council on Animal Care.

### Morpholino knockdowns and mRNA rescue

The following morpholinos (MOs) were purchased from Gene Tools:

*PrP2-MO1* 5′-ATTGTTAAGCGACCCATCTTTGGGC -3′

*PrP2-MO2*: 5′-CCAAGGGACAACAATCGCCCAAGAG-3′

They are designed to target respectively the start codon (*PrP2*-MO1) and the 5′ UTRs (*PrP2*-MO2) of zebrafish *prnprs3* gene [14].

The two MOs were delivered to 1- or 2-cell stage zebrafish embryos at 10 ng dose, with or without *p53* morpholino (5′-GCG CCA TTG CTT TGC AAG AAT TG-3′). A standard control MO (5′-CCT CTT ACC TCA GTT ACA ATT TAT A-3′) was delivered as control conditions. In rescue experiments, 100 to 500 pg of *PrP2* mRNA was included in the injection solution, synthesized from a expression plasmid containing full length *prnprs3* cDNA were (Genbank accession # NM_001013298, ZFIN ID: ZDB-GENE-041221-3), provided by RZPD consortium, referred as *PrP2*.

All microinjections were performed at early cleavage stages (one- to two-cell stage) using a manual micromanipulator (Narishige) coupled to a Transjector 5246 (Eppendorf). After running specificity and dose-dependency controls, *PrP2*-MO1 and *PrP2*-MO2 morpholinos were injected at a concentration of 1 mM (10 ng) and 0.5 mM (5 ng) respectively in Danieau buffer (58 mM NaCl, 0.7 mM KCl, 0.4 mM MgSO4, 0.6 mM Ca(NO3)2, 5.0 mM HEPES [pH 7.6]) and 0.125% Phenol Red (Sigma); both *PrP2*-MO1 and *PrP2*-MO2 morpholinos produced the same phenotype.

For rescue experiments, morpholinos in Danieau buffer were coinjected with capped mRNAs from 100 to 500 pg/nl at a 1∶1 ratio in 0.05 M KCl and 0.125% Phenol Red. At least 150 embryos were microinjected per experiment (1 nl injection volume) and kept at 28.8°C; analysis of phenotypes was carried out for 100 embryos per experiment. Phenotypes were photographed with an Axiocam camera on an Axioplan 2 microscope. Images were further processed with Image J.

### Fish strains and embryos

Zebrafish (*Danio rerio*) were maintained according to standard procedures [42]. Embryos were obtained from natural spawning and incubated in tank water at 28°C. *PrP2* mutant line was established as previously described [18]. Ganglia and central nervous system were visualized with the *nbt-dsred* line [43], the neuromasts and interneuromastic cells were observed with the *Tg(–8.0claudinB-GFPB:lynEGFP)^zf106^* line referred to as *claudinB-GFP* [44], hair cells were counted using *Brn3c::GFP* [45], Schwann cells were followed using *sox10-GFP* line [46]. Crossing heterozygous or homozygous adults generated embryos. Experiments were designed with the minimum number of animals necessary to produce meaningful results.

### PrP antibodies

Sequence alignments for zebrafish and mammal proteins reveal a high conserved homology for one region (126–160 aa), we tested 6 different clones targeting this region: SAF 32, 60, 61, 84, 11C6, 70. Only one clone gave a specific signal, the clone SAF84 (SPI-Bio, Massy, France), which has been obtained from Scrapie Associated Fibrils (SAF) purified from hamster brain infected and known to recognize the human proteins PrP^C^ and PrP^Sc^. The target epitope corresponds to the 126–164 human aa sequence (Fig S1) [29].

### Immunohistochemistry

Embryos were anaesthetized by immersion in 0.2 mg/ml MS222, fixed in 4% PFA and processed for wholemount antibody staining. Immunohistochemistry was performed according to standard protocols (Zebrafish book protocols), using mouse anti-PrP SAF84, SAF 32, 60, 61, 70, 11C6, mouse anti-E-cadherin (diluted 1∶500, Sigma), ß-catenin antibody (dilution 1∶100), MBP antibody (dilution 1∶200, ThermoScientific) and a secondary antibody conjugated to Cy3 (dilution 1∶800, Jackson Labs), Phalloidin-TRITC (dilution 1∶1000, Sigma). The samples were observed under confocal microscopy (Zeiss LSM 510, Leica SPE). Image stacks were processed with ImageJ.

### Alkaline Phosphatase staining

72 hpf-wild type AB strain zebrafish and *prp2* mutants were fixed in 4% PFA with 5% sucrose for 3–3.5 hours at room temperature. They were subsequently washed 4× in PBS/0.1% tween (PBST) and then for 15 minutes in fresh alkaline phosphatase buffer (100 mM Tris-HCl, 100 mM NaCl, 50 mM MgCl2) with 0.1% tween. They were developed in alkaline phosphatase buffer containing 0.225% NBT and 0.175% BCIP (Roche catalogue numbers 11383213001 and 11383221001, respectively) for approximately 10 minutes. Fish were then washed for 30 minutes in alkaline phosphatase wash buffer (154 mM NaCl, 11 mM Tris/HCl, 1 mM EDTA) with 0.1% tween, fixed in 4% PFA with 5% sucrose, washed 3× in PBST and imaged with a Leica M165 FC dissecting microscope and Leica DFC 400 camera.

### Western blot

Five dechorionated and deyolked [47] embryos were homogenized in extraction buffer (125 mM Tris-HCl pH 6.8, 4% SDS, 20% glycerol) with protease and phosphatase inhibitors (Roche, France). Twenty microliters of homogenate were separated on 10% SDS-polyacrylamide gels and transferred to nitrocellulose membranes. For antibody revelation, nitrocellulose membranes were incubated in Tris-buffered saline with 0.1% Tween 20 (TBST) supplemented with 5% skim milk powder for one hour and then soaked overnight at 4°C in TBST supplemented with 5% skim milk powder with the appropriate antibody, anti-PrP SAF84 (dilution 1∶1000) or anti-H2B (Abcam, UK, at 1∶1000) Immunosignals were visualized with anti-mouse or anti-rabbit IgG antibodies conjugated to horseradish peroxidase at 1∶2000 dilution (Sigma, France) and revealed with Luminata Crescendo Western HRP substrate (Millipore, France). Detection was done on the LICOR Odyssey and proteins bands were quantified with the NIH ImageJ sofware. At least three separate experiments were analysed and a ratio of PrP2 to H2B was then determined.

### Time-Lapse Imaging

Embryos were anesthetized in 0.01% tricaine (Sigma) and embedded in 1% agarose. Time-lapse analysis was carried out on a Zeiss microscope (Axioplan 2) and pictures were taken every 10 minutes between 25 and 45 hpf.

### Statistical analysis

Statistical analysis was performed using non-parametric tests (Kruskal-Wallis and Mann-Whitney tests) with Prism software.

## Supporting Information

Figure S1 **Alignments between human and zebrafish** ***PrP2*** **sequences: preferential sequence targeted by different monoclonal antibodies**. The region between 126-164 amino acids of the human PrP2 corresponds to one of the two highest homolog regions. Prion domains are represented: signal peptide (SP, blue), repetitive region (RP, yellow), hydrophobic domain (HD, red), globular domain (GD, cyan), GPI-anchored peptide (GPI, gray).
(TIF)

Movie S1 **Confocal z-stack of a control AB embryo at 30 hpf. Following DAPI nuclei/phalloidin-TRITC staining, primordium shape and two rosette are prominent.** Same control as presented in Fig. 4.
(MOV)

Movie S2 **Confocal z-stack of a control AB embryo at 30 hpf.** Following DAPI nuclei/phalloidin-TRITC staining, primordium shape and three rosette are prominent.
(MOV)

Movie S3 **Confocal z-stack of a** ***Prp2^−/−^*** **mutant at 30 hpf.** Following DAPI nuclei/phalloidin-TRITC staining, the primordium is disorganized; only one actin concentration point is visible. Same *Prp2^−/−^* mutant as presented in Fig. 4.
(MOV)

Movie S4 **Confocal z-stack of a** ***Prp2^−/−^*** **mutant at 30 hpf.** Following DAPI nuclei/phalloidin-TRITC staining, primordium edges are visible and two apical rosette centers are present however actin staining is not as strong as control.
(MOV)

Movie S5 **Confocal z-stack of a** ***Prp2^−/−^*** **mutant at 30 hpf.** Following DAPI nuclei/phalloidin-TRITC staining, the primordium is more elongated and disorganized; only one actin concentration point is visible.
(MOV)

Movie S6 **Control embryo** ***claudinB-GFP*** **at 30 hpf.** The primordium migrates along the myoseptum at a regular speed.
(MOV)

Movie S7 ***PrP2*** **-MO embryo** ***claudinB-GFP*** **at 30 hpf.** The primordium migrates at an irregular speed, becomes rounded, stopped and finally migrate a little further.
(MOV)
