# Supplementary figures and images for: Zebrafish Prion Protein PrP2 Controls Collective Migration Process during Lateral Line Sensory System Development
Source: PLoS One. 2014 Dec 1;9(12):e113331. doi: 10.1371/journal.pone.0113331 (PMC4249873; doi:10.1371/journal.pone.0113331)

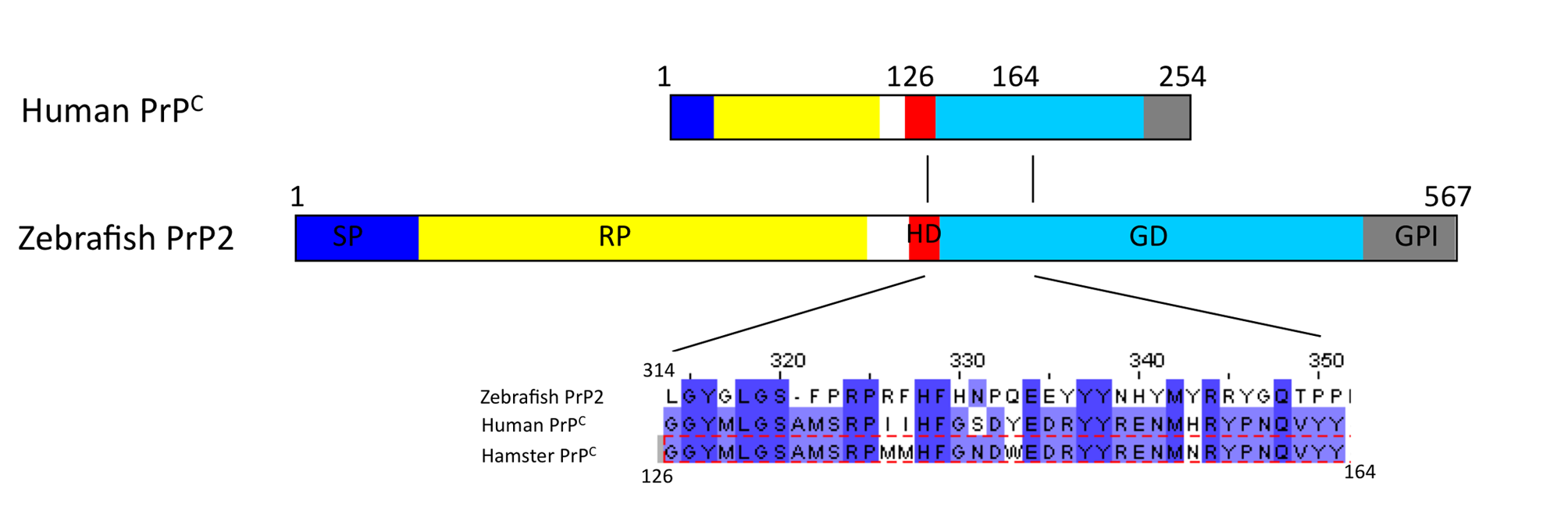

Supplement: Figure S1 — Alignments between human and zebrafish PrP2 sequences: preferential sequence targeted by different monoclonal antibodies. The region between 126-164 amino acids of the human PrP2 corresponds to one of the two highest homolog regions. Prion domains are represented: signal peptide (SP, blue), repetitive region (RP, yellow), hydrophobic domain (HD, red), globular domain (GD, cyan), GPI-anchored peptide (GPI, gray). (TIF) [file pone.0113331.s001.tif]
